# Accurate diagnosis of Alzheimer’s disease using specific volatile organic compounds from breath

**DOI:** 10.1002/alz.087440

**Published:** 2025-01-09

**Authors:** Lu Shen, Sizhe Zhang, Haokun Liu, Ziyu Ouyang, Tianyan Xu, Qijie Yang, Yuan Zhu, Meidan Wan, Xuewen Xiao, Xuan Yang, Li Yuan, Yuzhang Bei, Junling Wang, Jifeng Guo, Haibin Chen, Beisha Tang, Bin Jiao

**Affiliations:** ^1^ Xiangya Hospital, Central South University, Changsha, Hunan China; ^2^ Jili Hospital, Changsha, Hunan China; ^3^ PCAB Research Center of Breath and Metabolism, Beijing, Beijing China

## Abstract

**Background:**

Existing biomarkers including cerebrospinal fluid and positron emission tomography for Alzheimer’s disease (AD) diagnosis are relatively invasive and expensive. Application of exhaled breath collection and volatile organic compound (VOC) detection for AD diagnosis remains unclear.

**Method:**

In this cross‐sectional study, high‐pressure photon ionization time‐of‐flight mass spectrometry (HPPI‐ToF‐MS) was used to detect VOCs from breath in three datasets and patients diagnosed as Parkinson’s disease (PD). Plasma p‐tau181 was quantified using ultra‐sensitive Simoa technology. GC x GC TOF‐MS was used to detect VOCs in plasma. Near‐infrared spectroscopy was carried out to elucidate alterations of brain metabolism.

**Result:**

A total of 861 participants were enrolled. Patients with AD were diagnosed according to the 2011 NIA‐AA diagnosis criteria and further confirmed in dataset 1 (n=30) based on the A+T+N+ framework. Datasets 1 and 2 were used to recognize the different VOCs between patients with AD and controls. The diagnostic power of VOCs for AD was confirmed separately in train datasets 1 and 2 and independent test dataset 3. The specific VOCs in AD diagnosis were further compared with patients with PD. Among all participants, no statistical difference was observed in demographic characteristics for dataset 1. Six VOCs, including ethanol (m/z = 46), isopropanol (m/z = 60), chloroethane (m/z = 64), pyrrole/3‐butenenitrile (m/z = 67), 1‐butanol (m/z = 74), and benzene (m/z = 78), were significantly different between the two groups in dataset 1 and were verified in dataset 2 and dataset 3. Ethanol (m/z=46) and pyrrole/3‐butenenitrile (m/z=67) presented areas under the ROC curve (AUCs) values of 0.907 and 0.895, respectively, in datasets 1 and 2 and 0.849 and 0.974, respectively, in dataset 3 (community). The six VOCs were associated with cognitive decline as reflected by neuropsychological tests, five (m/z = 46, 64, 67, 74, and 78) were correlated with plasma p‐tau181 levels, and five (m/z = 46, 60, 67, 74, and 78) showed consistent alteration in plasma. Moreover, five (m/z = 46, 60, 67, 74, and 78) were associated with altered brain connectivity.

**Conclusion:**

Specific breath VOCs may be used as simple biomarkers for AD detection.

